# FSTL3 is associated with prognosis and immune cell infiltration in lung adenocarcinoma

**DOI:** 10.1007/s00432-023-05553-w

**Published:** 2024-01-19

**Authors:** Xiangzhi Meng, Xiaojian Zhao, Boxuan Zhou, Weijian Song, Yicheng Liang, Mei Liang, Minjun Du, Jianwei Shi, Yushun Gao

**Affiliations:** 1 Department of Thoracic Surgery, National Cancer Center/National Clinical Research Center for Cancer/Cancer Hospital, Chinese Academy of Medical Sciences and Peking Union Medical College, Chaoyang District, Panjiayuan, Nanli 17, Beijing, 100021 People’s Republic of China; 2 Department of Thoracic Surgery, Sun Yat-Sen Memorial Hospital, Sun Yat-Sen University, 107 Yanjiang West Road, Guangzhou, 510120 China

**Keywords:** FSTL3, Lung adenocarcinoma, Prognosis, Immune cell infiltration

## Abstract

**Purpose:**

FSTL3 expression is altered in various types of cancer. However, the role and mechanism of action of FSTL3 in lung adenocarcinoma development and tumor immunity are unknown. We investigated the association between FSTL3 expression and clinical characteristics and immune cell infiltration in lung adenocarcinoma samples from The Cancer Genome Atlas (TCGA) and a separate validation set from our hospital.

**Methods:**

Data on immune system infiltration, gene expression, and relevant clinical information were obtained by analyzing lung adenocarcinoma sample data from TCGA database. Using online tools like GEPIA, the correlations between FSTL3 expression and prognosis, clinical stage, survival status, and tumor-infiltrating immune cells were examined. In a validation dataset, immunohistochemistry was performed to analyze FSTL3 expression and its related clinical characteristics.

**Results:**

FSTL3 expression was markedly reduced in patients with lung adenocarcinoma. N stage, pathological stage, and overall survival were significantly correlated with FSTL3 expression. According to GSEA, FSTL3 is strongly linked to signaling pathways such as DNA replication and those involved in cell cycle regulation. Examination of TCGA database and TIMER online revealed a correlation between FSTL3 and B cell, T cell, NK cell, and neutrophil levels. The prognosis of patients with lung adenocarcinoma was significantly affected by six genes (*KRT6A*, *VEGFC*, *KRT14*, *KRT17*, *SNORA12*, and *KRT81*) related to FSTL3.

**Conclusion:**

FSTL3 is significantly associated with the prognosis and progression of lung adenocarcinoma and the infiltration of immune cells. Thus, targeting FSTL3 and its associated genes in immunotherapy could be potentially beneficial for the treatment of lung adenocarcinoma.

**Supplementary Information:**

The online version contains supplementary material available at 10.1007/s00432-023-05553-w.

## Introduction

Lung cancer is a highly prevalent and deadly form of malignant tumor (Bray et al. 2018). Non-small cell lung cancer (NSCLC), which comprises approximately 80–85% of all lung cancer cases, can be further categorized into two main subtypes: lung adenocarcinoma (LUAD) and lung squamous cell carcinoma. LUAD accounts for approximately 40% of all lung cancers and is the most prevalent form of lung cancer. Despite the development of treatments to enhance patient outcomes, prognosis remains unfavorable for patients with LUAD. The 5-year survival rate of patients with advanced cancer is 10–15% (Kim et al. 2020; Siegel et al. 2012). Currently, surgery, radiotherapy, chemotherapy, and targeted therapy are the primary therapeutic approaches for LUAD. However, these methods insufficiently improve the symptoms and outcomes of patients with LUAD and survival rates. Hence, continuous exploration of novel and promising molecular indicators is imperative to identify targets to enhance the survival and prognosis of patients with LUAD.

FSTL3 belongs to the follistatin-like protein family. The *FSTL3* gene contains five exons and is located on chromosome 19p13. FSTL3 is expressed in various tissues throughout the human body, including the heart, placenta, gonads, and pancreas. The regulation of FSTL3 mRNA transcription is controlled by transforming growth factors and is indirectly enhanced by activin-A via SMAD family proteins (Huret et al. 2001). FSTL3 can affect myocyte development by directly inhibiting myogenin and activin-A through SMAD-dependent mechanisms (Nam et al. 2015; Smith and Lin 2013).

Typically, FSTL3 inhibits the activities of activin-A and myostatin (Brandt et al. 2014). Nevertheless, there have been limited reports on FSTL3 expression and its role in tumors, aside from its regulation of biological functions. The expression of FSTL3 in hepatocellular carcinoma tissues is lower than that in normal liver tissues (Grusch et al. 2006). Although breast cancer tissue and serum have shown high levels of FSTL3 (Bloise et al. 2009; Li et al. 2017), it is not significantly associated with patient prognosis (Couto et al. 2017). A study on lung cancer revealed that FSTL3 expression is elevated in both NSCLC tissues and cell lines. These increased levels are associated with poor prognoses in patients with NSCLC (Gao et al. 2020). Nevertheless, the expression of FSTL3 in LUAD and its influence on prognosis remains unclear.

Using The Cancer Genome Atlas (TCGA) database and clinical samples from our hospital, we discovered that FSTL3 holds significant prognostic significance and is linked to the infiltration of immune cells in individuals with LUAD. We also investigated the predictive significance of molecular controls and genes associated with FSTL3 in individuals diagnosed with LUAD, offering novel insights and avenues for prognostic prediction in patients with this disease.

## Methods

### TCGA data

The RNA-seq data for the STAR process of TCGA-LUAD project were obtained from TCGA database (https://portal.gdc.cancer.gov). The data in TPM format were extracted and clinical data were collected. Subsequently, the LUAD data in TCGA database were utilized to conduct gene expression, immune infiltration, and clinical-related information analyses. The analysis included 59 normal and 539 tumor tissue samples and prognostic data from previous studies (Liu et al. 2018). Differential gene analysis was conducted in LUAD groups with high and low FSTL3 expression. TNM staging of LUAD cases was determined using the staging: eighth edition from the American Joint Committee on Cancer (AJCC). A Kaplan–Meier survival analysis was conducted using the expression levels of FSTL3 and the survival status of patients with LUAD. The 539 LUAD tumor samples were categorized into two groups based on FSTL3 expression, and the corresponding clinical characteristics of the patients in each group are shown in Supplementary Table 1.

### External validation with hospital data

A retrospective analysis of 100 patients diagnosed with LUAD was conducted at the Cancer Hospital of the Chinese Academy of Medical Sciences between January 2017 and December 2018. The patients included in the study met the following criteria: (i) they had undergone surgery at the hospital, (ii) LUAD was confirmed through postoperative pathology, and (iii) complete clinical and follow-up data were available. Patients with other malignant tumors, different types of lung cancer confirmed by postoperative pathology, missed visits, or incomplete clinical data were excluded. Following the screening process, a total of 94 patients were included in this study; four patients who were lost to follow-up and two who declined to participate were excluded. The Ethics Committee of the Cancer Hospital of the Chinese Academy of Medical Sciences approved the acquisition of tissue samples and clinical data (Approval No: NCC2019C-167). Tumor tissues from 94 patients were subjected to paraffin sectioning and immunohistochemical staining. Throughout this investigation, every registered individual was monitored for 5 years after or until death. Approval of the study methodology and signing of the informed consent statement by all patients enrolled in the study.

### Immunohistochemical staining and analysis

Following the removal of paraffin and restoration of moisture, the tissue sections were sealed using a solution containing 3% hydrogen peroxide. The process of repairing antigens damaged by heat was performed using Tris–EDTA buffer at pH 9.0. The sections were exposed to the primary antibody at a temperature of 4 ℃ overnight, followed by washing with phosphate-buffered saline. Subsequently, the cells were treated with polyclonal FSTL3 antibody (HPA045378; Sigma-Aldrich) for 30 min, followed by incubation with horseradish peroxidase-polymer antibody for 30 min. A color development reaction was induced by adding 3,3'-diaminobenzidine. The sections were treated with hematoxylin, dehydrated, and sectioned; images of the stained sections were taken, and 10 regions were randomly chosen from each image. Positive regions showing FSTL3 expression were analyzed using Image Pro Plus software (version 6.0). The gray value of each region was recorded, and the average value was calculated as the FSTL3 expression level.

### Gene set enrichment analysis (GSEA)

GSEA utilizes a predetermined gene dataset to analyze distribution patterns in a gene table, sorted based on phenotypic significance. This analysis helps to determine the contribution and significance of these genes to the phenotype. To investigate the role of FSTL3 in molecular functions (MF), biological processes (BP), intracellular components, and cellular pathways, we examined data from TCGA for GO and KEGG pathway enrichment using the R language software (Yu et al. 2012).

### Examining immune cell infiltration in tumors

TIMER (https://cistrome.shinyapps.io/timer/) was used to analyze genomic variation to confirm the correlation between FSTL3 expression and 24 immune cell markers. Methods can refer to the previously published article (Li et al. 2016). To validate significant and pertinent genes identified in TIMER, the GEPIA tool (http://gepia.cancer-pku.cn/?from=timeline&isappinstalled=0) was used. To confirm the correlation between FSTL3 expression and 24 immune cell markers (Bindea et al. 2013), we performed genomic variation analysis (Hänzelmann et al. 2013).

### Statistical analysis

Data type variables were analyzed using Student’s *t*-test. Continuous variables were analyzed using the chi-square test. The clinical characteristics of the two groups were compared using either the chi-square or Fisher's exact tests. Data were analyzed using R (version 4.2.1), and proportional risk hypothesis testing was conducted using Cox regression analysis with the survival package. Samples that met a specified *p*-value threshold for a single factor were used to build the multifactor Cox model. The survival package was used to conduct hypothesis testing based on the proportional risk hypothesis, and survival regressions were fitted. Additionally, the Survminer and ggplot2 packages were used to visualize the results. Statistical significance was set at *p* < 0.05.

## Results

### FSTL3 expression in LUAD

**Fig. 1:**
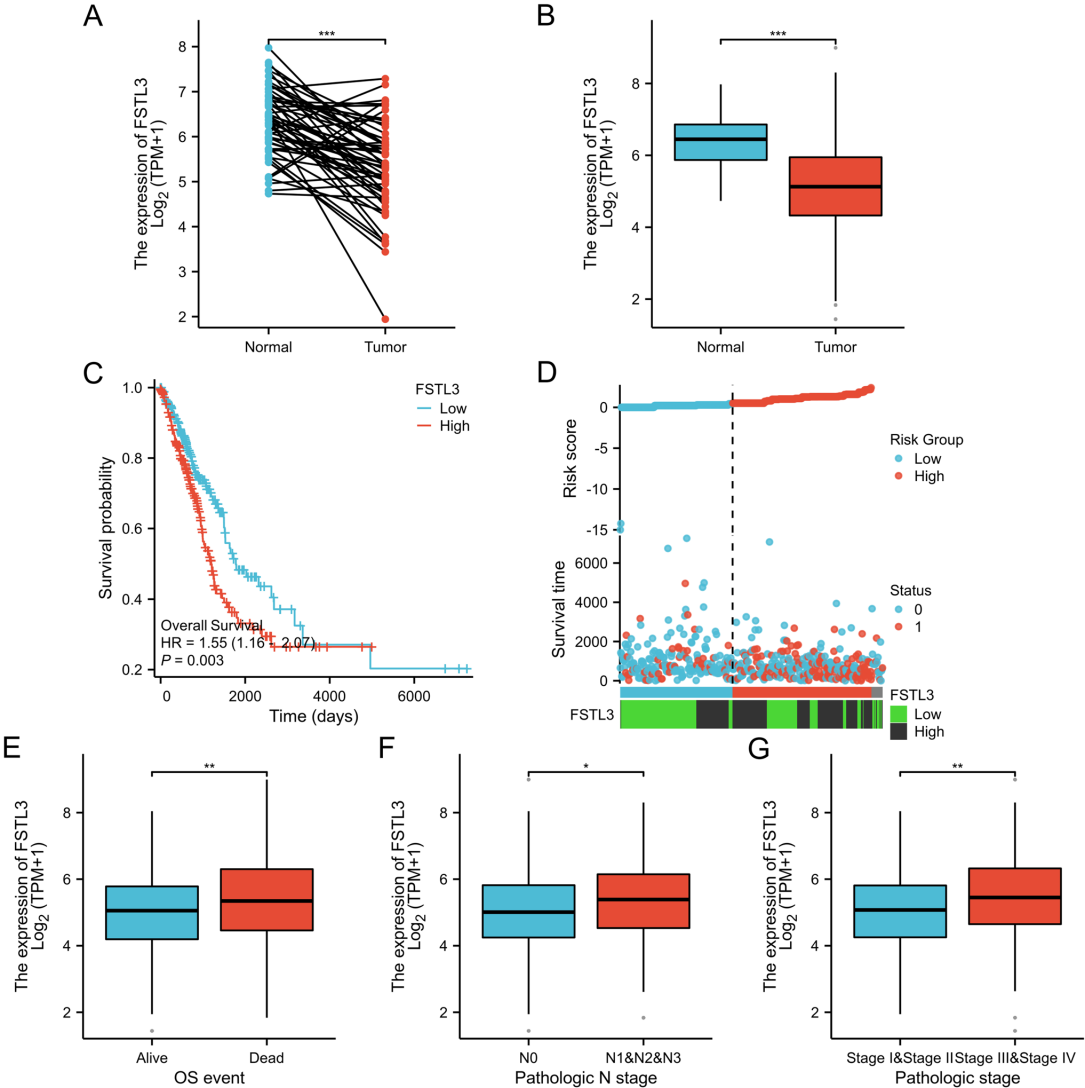
Comparison of the expression of FSTL3 in normal and LUAD tumor tissues. Analysis of FSTL3 expression in normal and LUAD tissues was conducted using paired (**A**) and unpaired (**B**) methods. Additionally, Kaplan–Meier survival curve analysis was performed to examine the impact of high and low FSTL3 expression on LUAD prognosis (**C**). The correlation between FSTL3 expression and survival status (0 for survival, 1 for death) (**D**). The relationship between FSTL3 expression and overall survival (OS) (**E**), metastatic status of lymph nodes (**F**), and pathological staging (**G**) was explored. Statistical significance was denoted as *p < 0.05, **p < 0.01, ***p < 0.001

The expression of FSTL3 in LUAD was examined by comparing both LUAD and normal tissue data from TCGA database. TCGA dataset contained 59 healthy tissue and 539 cancerous tissue sample data. The findings indicated that the FSTL3 expression level in tumor tissues was considerably lower than that in normal tissues (*p* < 0.001) in both paired (Fig. 1A) and unpaired (Fig. 1B) examinations.

The correlation between FSTL3 expression level and overall survival (OS) was assessed in individuals diagnosed with LUAD. The results indicated a significant correlation between the prognosis of patients with LUAD and the expression level of FSTL3 (HR = 1.55, 95%CI 1.16–2.07, *p* = 0.003), as demonstrated in Fig. 1C. The findings from the analysis of the GEPIA online database (Supplementary Fig. 1) confirmed these findings, and we examined the association between clinical variables and OS. FSTL3 expression, pathologic stage, T stage, and N stage were significantly associated with OS (*p* < 0.05) (Table 1). Additionally, multifactorial analysis revealed that Stage IV, T3, and N1 were associated with poor prognosis (Table 1). The correlations between FSTL3 expression, survival status, and hazard scores were examined in Fig. 1D. Patients in the high hazard score group exhibited elevated levels of FSTL3 expression and improved survival compared to those in the low hazard score group.

**Table 1:** Correlation between univariate and multivariable characteristics in LUAD patients

Characteristics	Total (*N*)	Univariate analysis		Multivariate analysis	
		Hazard ratio (95% CI)	*P* value	Hazard ratio (95% CI)	*P* value
Age	520				
< = 65	257	Reference			
> 65	263	1.216 (0.910–1.625)	0.186		
Gender	530				
Female	283	Reference			
Male	247	1.087 (0.816–1.448)	0.569		
Pathologic stage	522				
Stage I	292	Reference		Reference	
Stage II	123	2.341 (1.638–3.346)	** < 0.001**	1.232 (0.663–2.291)	0.509
Stage III	81	3.576 (2.459–5.200)	** < 0.001**	1.828 (0.731–4.572)	0.197
Stage IV	26	3.819 (2.211–6.599)	** < 0.001**	2.405 (1.152–5.021)	**0.019**
Pathologic T stage	527				
T1	176	Reference		Reference	
T2	285	1.507 (1.059–2.146)	**0.023**	1.255 (0.872–1.807)	0.222
T3	47	2.964 (1.762–4.986)	** < 0.001**	2.168 (1.156–4.065)	**0.016**
T4	19	3.357 (1.767–6.376)	** < 0.001**	1.363 (0.651–2.851)	0.411
Pathologic N stage	514				
N0	345	Reference		Reference	
N1	96	2.293 (1.632–3.221)	** < 0.001**	1.804 (1.008–3.228)	**0.047**
N2 and N3	73	2.993 (2.057–4.354)	** < 0.001**	1.569 (0.681–3.615)	0.290
FSTL3	530				
Low	266	Reference		Reference	
High	264	1.550 (1.160–2.070)	**0.003**	1.343 (0.993–1.815)	0.055

The bold values means the *P*-value for comparison of the two groups was less than 0.05 and statistically significant

In patients with LUAD, an additional analysis of the association was conducted between FSTL3 expression and clinicopathological factors, and the results indicated a correlation between decreased expression of FSTL3 and OS (Fig. 1E), pathologic N stage (Fig. 1F), and pathological stage (Fig. 1G) (stage III and stage IV vs stage I and stage II, *p* = 0.023) (Table 2). These findings indicated that patients with higher FSTL3 expression had worse survival rates and clinicopathological stages.

**Table 2:** Association between FSTL3 expression and clinicopathologic characteristics using logistic regression

Characteristics	Total (*N*)	OR (95% CI)	*P* value
Pathologic T stage (T1 and T2 vs. T3 and T4)	536	0.665 (0.397–1.114)	0.121
Age (> 65 vs. < = 65)	520	1.222 (0.866–1.724)	0.254
Gender (Male vs. Female)	539	0.908 (0.647–1.274)	0.577
Pathologic N stage (N0 vs. N1 and N2 and N3)	523	0.600 (0.415–0.867)	**0.007**
Pathologic M stage (M0 vs. M1)	390	0.790 (0.349–1.786)	0.571
Pathologic stage (Stage III and Stage IV vs. Stage I and Stage II)	531	1.642 (1.072–2.516)	**0.023**

The bold values means the *p*-value for comparison of the two groups was less than 0.05 and statistically significant

### Differentially expressed genes and gene enrichment analysis

**Fig. 2:**
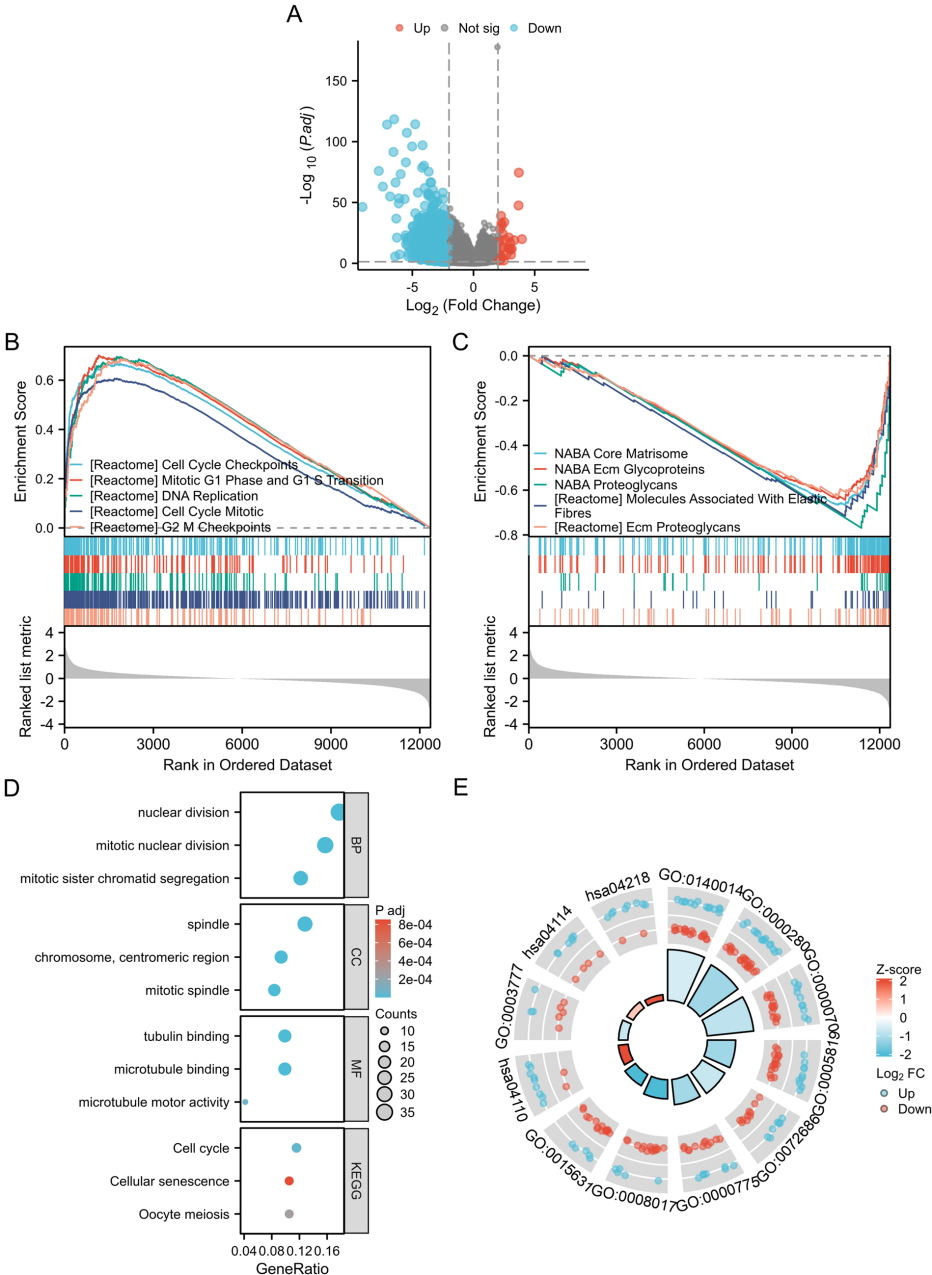
GO and KEGG analysis of genes related to FSTL3, and the top 5 pathways from GSEA that showed positive and negative correlations. **A** Volcano plots of the differential genes between high and low expression of FSTL3 in LUAD tissues. GSEA revealed the top 5 pathways that were positively correlated (**B**) and negatively correlated with FSTL3 expression (**C**). **D** GO and KEGG analysis of differentially expressed genes related to high and low FSTL3 expression; **E** Combined logFC enrichment analysis for GO and KEGG analysis results

As shown in Fig. 2A, 3481 genes were differentially expressed between the FSTL3 high- and low-expression groups, with 30 genes that were upregulated and 3451 genes that were downregulated. After, the possible biological roles of FSTL3 were examined through GSEA. GSEA analysis showed that a total of 596 pathways associated with FSTL3 genes were enriched (|NES|) > 1, *p* < 0.05). This included 441 pathways that showed a positive correlation (NES > 1) and 155 pathways that showed a negative correlation (NES <  − 1). Table 3 shows the five most significant pathways, both positive and negative, selected according to the normalized enrichment score (NES). The five pathways that were positively correlated with FSTL3 expression were CELL_CYCLE_CHECKPOINTS, MITOTIC_G1_PHASE_AND_G1_S_TRANSITION, DNA_REPLICATION, CELL_CYCLE_MITOTIC, and G2_M_CHECKPOINTS. The pathways that showed a negative correlation with FSTL3 expression were ECMPROTEOGLYCANS, MOLECULESASSOCIATEDWITHELASTICFIBRES, PROTEOGLYCANS, ECMGLYCOPROTEINS, and COREMATRISOME (Fig. 2B and C, and Table 3). Differentially expressed genes between the two groups with high and low FSTL3 expression were analyzed using GO and KEGG analysis. The results, based on the hypergeometric distribution, revealed significant enrichment in BP related to nuclear division, cellular components (CC) associated with spindles, and MF involving microtubule binding. Additionally, KEGG analysis indicated significant enrichment of the cell cycle pathway (Fig. 2D). Figure 2E shows the combined visualization of GO and KEGG results, along with logFC enrichment analysis.

**Table 3:** Signaling pathways most significantly correlated with FSTL3 expression

	Signaling pathways	NES	*P* value
Positive	CELL_CYCLE_CHECKPOINTS	3.043	< 0.001
	MITOTIC_G1_PHASE_AND_G1_S_TRANSITION	3.002	< 0.001
	DNA_REPLICATION	2.956	< 0.001
	CELL_CYCLE_MITOTIC	2.947	< 0.001
	G2_M_CHECKPOINTS	2.901	< 0.001
Negative	ECM_PROTEOGLYCANS	− 2.239	< 0.001
	MOLECULES_ASSOCIATED_WITH_ELASTIC_FIBRES	− 2.267	< 0.001
	PROTEOGLYCANS	− 2.361	< 0.001
	ECM_GLYCOPROTEINS	− 2.534	< 0.001
	CORE_MATRISOME	− 2.752	< 0.001

### Correlation between FSTL3 and tumor-associated immune infiltrating cells in LUAD

**Fig. 3:**
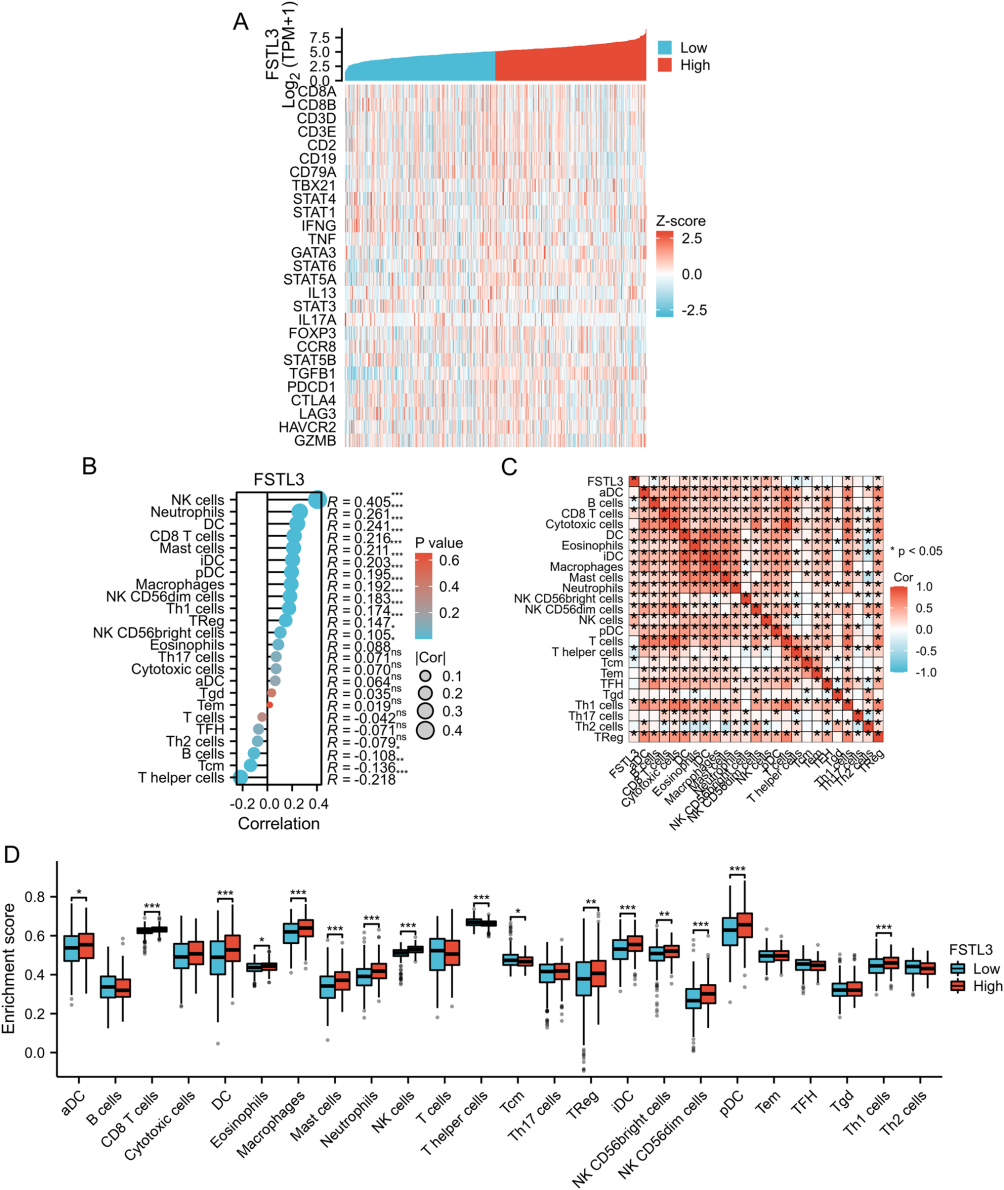
Associations between FSTL3 expression and immune cells, as well as genes related to immune cells. **A** Heatmap displaying the relationship between FSTL3 expression and various genes related to immune cells. **B** The correlation between FSTL3 and immune cells in LUAD tissues. **C** Heatmap illustrating the presence of infiltrating immune cells in LUAD tissues. **D** Comparative analysis of 24 subtypes of immune cells between the high- and low-expression FSTL3 groups. *p*-values less than 0.05 are denoted as *p* < 0.05, *p*-values less than 0.01 are denoted as **p < 0.01, and *p*-values less than 0.001 are denoted as ***p < 0.001

Using the TIMER online database, the correlation between FSTL3 expression and immune cell infiltration in tumor tissues was examined and found that the expression of FSTL3 was strongly correlated with B lymphocytes (*p* < 0.001), CD4 + T lymphocytes (*p* = 0.009), macrophages (*p* = 0.01), and neutrophils (*p* < 0.001) (Supplementary Fig. 2). Analysis of TCGA database showed a significant correlation between the expression of FSTL3 and NK cells, neutrophils, and CD8 + T cells (Fig. 3B), which was partially supported by previous findings.

The findings indicate a correlation between FSTL3 expression and the infiltration of immune cells in LUAD. Next, we looked at whether high and low FSTL3 expression had the same effects on the tumor immune microenvironment. We assessed the differences in the levels of 24 immune cell types between the two groups, including T cells (such as Th1 cells, Treg, CD8 + T cells), DCs, Macrophages, Neutrophils, NK cells, and others. The immune cell subtypes that were correlated with FSTL3 expression are illustrated in Fig. 3D and Table 4. Figure 3C displays a correlation heatmap illustrating the connections among the levels of various immune cell subtypes in LUAD tissues.

**Table 4:** Correlation analysis between IL17A and immune cells

Immune cells	Pearson		Spearman	
	COR	*P* value	COR	*P* value
aDC	0.096	0.024	0.063	0.140
B cells	− 0.087	0.042	− 0.108	0.011
CD8 T cells	0.260	0.000	0.216	0.000
Cytotoxic cells	0.089	0.037	0.069	0.104
DC	0.261	0.000	0.240	0.000
Eosinophils	0.107	0.012	0.088	0.040
iDC	0.206	0.000	0.202	0.000
Macrophages	0.198	0.000	0.192	0.000
Mast cells	0.213	0.000	0.211	0.000
Neutrophils	0.264	0.000	0.261	0.000
NK CD56bright cells	0.153	0.000	0.105	0.014
NK CD56dim cells	0.200	0.000	0.182	0.000
NK cells	0.413	0.000	0.405	0.000
pDC	0.201	0.000	0.194	0.000
T cells	− 0.042	0.320	− 0.042	0.324
T helper cells	− 0.248	0.000	− 0.217	0.000
Tcm	− 0.196	0.000	− 0.136	0.001
Tem	− 0.001	0.976	0.019	0.653
TFH	− 0.066	0.122	− 0.070	0.101
Tgd	0.045	0.294	0.034	0.418
Th1 cells	0.180	0.000	0.174	0.000
Th17 cells	0.104	0.015	0.071	0.098
Th2 cells	− 0.055	0.196	− 0.078	0.067

Subsequently, the association between FSTL3 expression and marker genes of various immune cells was investigated. These findings indicate a connection between the expression of FSTL3 and markers of B lymphocytes and various forms of T lymphocytes (Fig. 3A).

### Correlations between FSTL3 expression and the expression of related genes

**Fig. 4:**
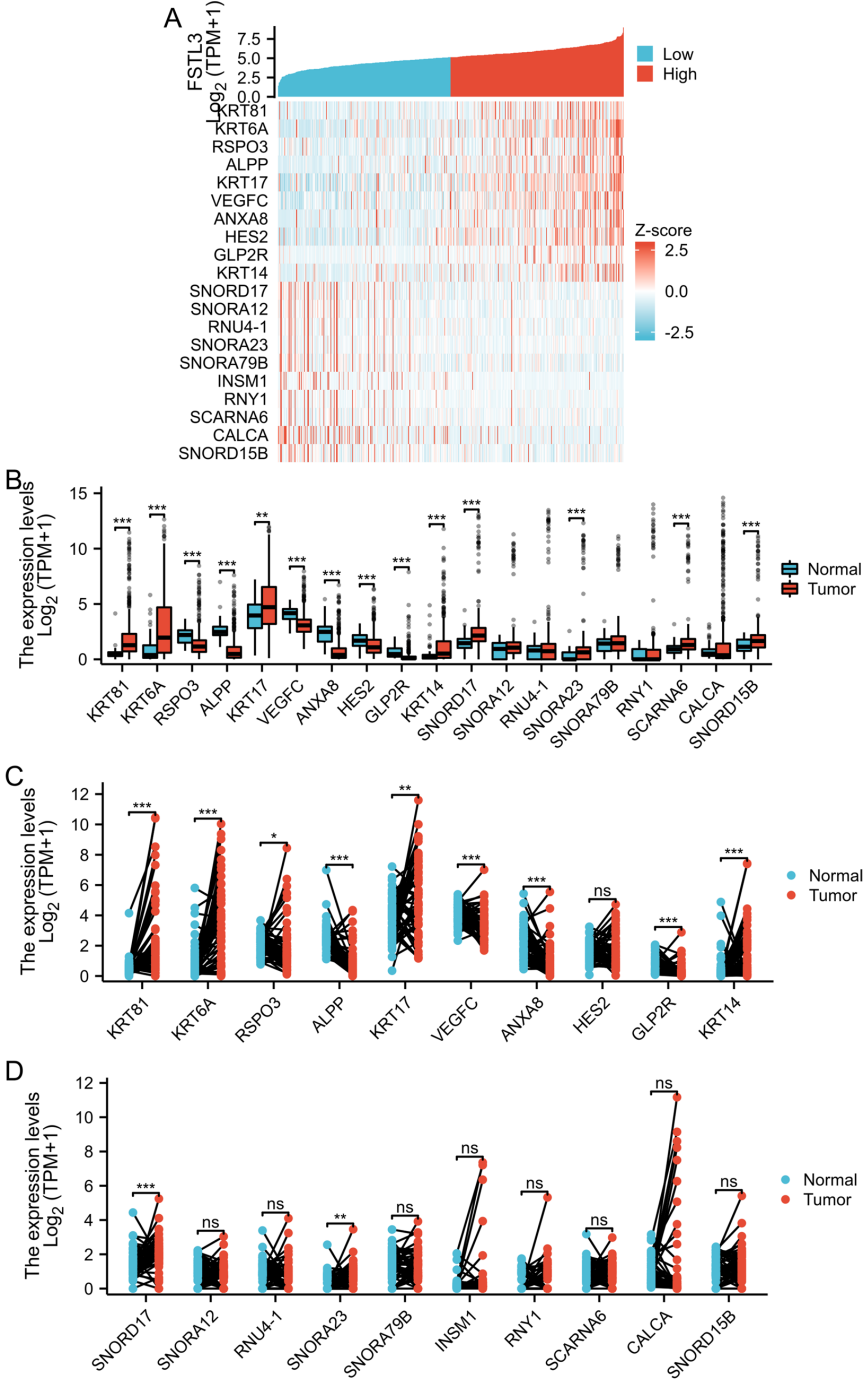
Heat maps were used to identify the top 10 genes that showed positive and negative correlations with FSTL3 expression in LUAD and normal tissues. Additionally, paired and unpaired analyses of associated genes were conducted. **A** Heatmap showing the expression pattern of the top 10 genes that were positively and negatively correlated with FSTL3 expression, using unpaired analysis. **B** Additionally, paired analysis was conducted for 20 genes associated with FSTL3 in both normal tissues and LUAD tissues. **C** represents positively correlated genes, while **D** represents negatively correlated genes. *p* values less than 0.05 are denoted as *p* < 0.05, *p* values less than 0.01 are denoted as **p < 0.01, and *p* values less than 0.001 are denoted as ***p < 0.001

Correlations, both positive and negative, between the expression of the top 10 genes and FSTL3 expression in LUAD are displayed in a correlation heatmap (Fig. 4A). TCGA dataset was analyzed to identify the correlations between the expression of the top 10 genes and FSTL3 expression, and both positive and negative correlations were observed in the LUAD and normal tissue samples. In the unpaired analysis, the *KRT81*, *KRT6A*, and *KRT17* genes, which were positively associated with FSTL3 expression, exhibited notably elevated expression in tumor tissues in comparison to that in normal tissues. The expression of *RSPO3*, *ALPP*, *VEGFC*, *ANXA8*, *HES2*, *GLP2R*, and *KRT14* in normal tissues was significantly higher than that in tumor tissues (Fig. 4B). Among the genes that showed a negative correlation with FSTL3 expression, *SNORD17*, *SNORA23*, *SCARNA6*, and *SNORD15B* exhibited significantly increased expression in tumor tissues compared to that in normal tissues. The expression levels of the remaining negatively correlated genes showed no significant differences (Fig. 4B). Pairwise analysis revealed that tumor tissues exhibited significantly elevated expression of *KRT81*, *KRT6A*, *KRT17*, and *KRT14*, which were positively correlated with FSTL3 expression, compared to that in normal tissues. Figure 4C shows that normal tissues exhibited notably higher expression levels of RSPO3, ALPP, VEGFCANXA8, and GLP2R in comparison to that in tumor tissues. Among the genes that had a negative correlation, only *SNORD17* and *SNORA23* exhibited a notable increase in expression in tumor tissues, which was statistically significant, as shown in Fig. 4D.

**Fig. 5:**
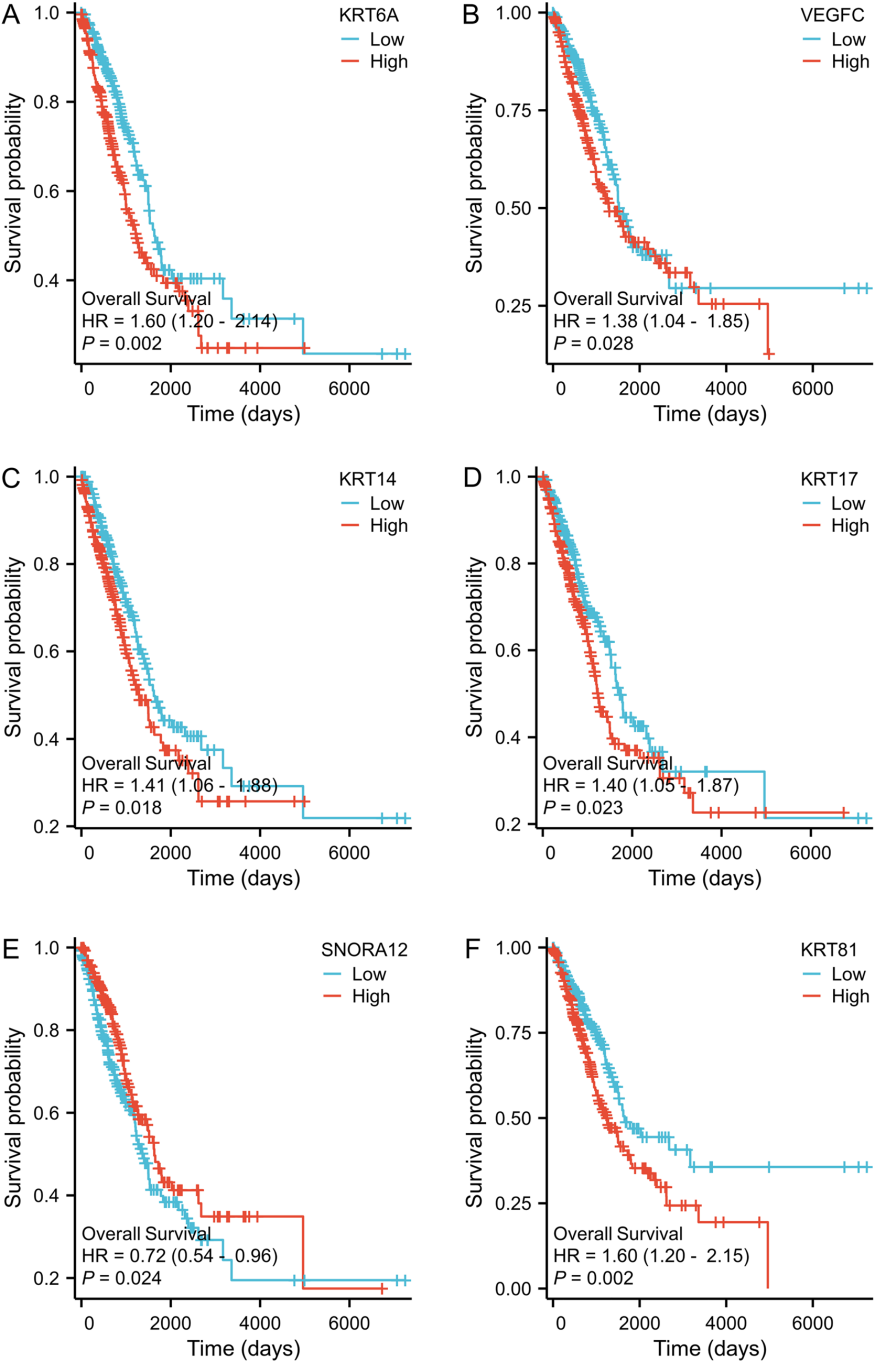
Survival curve analysis (OS) for six genes linked to FSTL3 expression in patients with LUAD: **A** impact of KRT6A gene expression on patient OS; **B** impact of VEGFC gene expression on patient OS; **C** impact of KRT14 gene expression on patient OS; **D** impact of KRT17 gene expression on patient OS; **E** impact of SNORA12 gene expression on patient OS; and **F** impact of KRT81 gene expression on patient OS

Prognostic analyses were conducted for the top 10 positively and negatively correlated genes associated with FSTL3 expression. The analyses revealed that alterations in the expression of six genes, KRT6A, VEGFC, KRT14, KRT17, SNORA12, and KRT81, had a notable influence on the prognosis of patients with LUAD (Fig. 5).

### External validation of the expression of genes correlated with centralized FSTL3

**Fig. 6:**
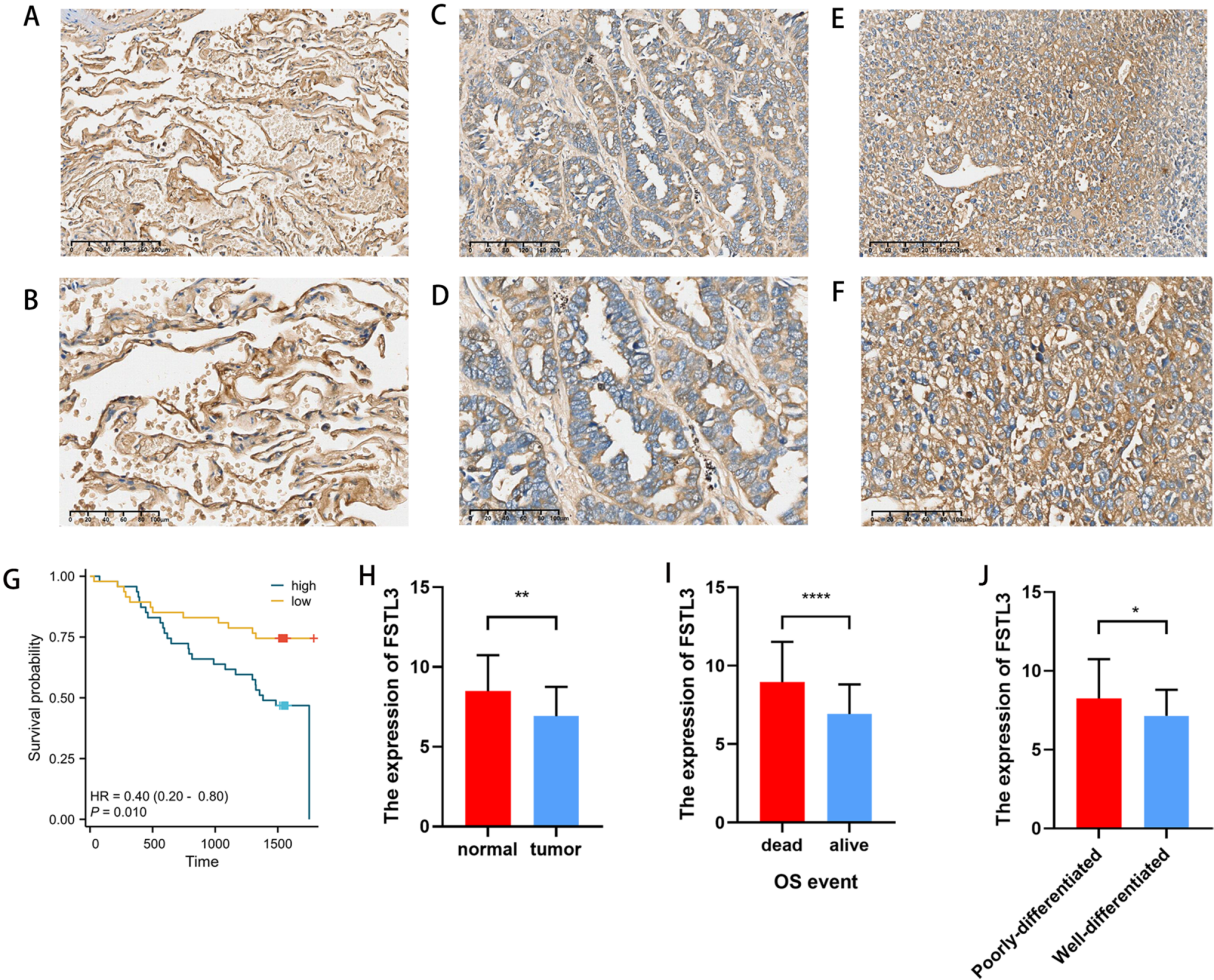
Associations with FSTL3 expression in the external validation dataset. Expression levels of FSTL3 in normal paracancerous tissue, imaged at 10× (**A**) and 20× (**B**); well-differentiated lung adenocarcinoma (LUAD) tissues, imaged at 10× (**C**) and 20× (**D**); poorly differentiated LUAD tissues, imaged at 10× (**E**) and 20× (**F**). The effect of high and low FSTL3 expression on overall survival (OS) in LUAD patients (**G**). Differences in FSTL3 expression between paracancerous and LUAD tissues (**H**). Differences in FSTL3 expression between surviving and dying patients with LUAD (**I**). Differences in FSTL3 expression between poorly differentiated and well-differentiated LUAD tissues (**J**). *p < 0.05, **p < 0.01, ***p < 0.001, ****p < 0.0001

In the external validation dataset from our center, immunohistochemistry analysis indicated a notable decrease in the expression of FSTL3 in LUAD tissues (*p* < 0.01) compared to that in adjacent normal tissues (Fig. 6A, B). Figure 6H illustrates the variations in the expression levels of FSTL3 across various degrees of differentiated cancer tissues. FSTL3 expression was higher in poorly differentiated LUAD tissues (Fig. 6E, F). In the more distinct LUAD tissues (Fig. 6C, D), the FSTL3 expression level was significantly reduced (*p* < 0.01; Fig. 6J). The correlation between FSTL3 expression levels and OS in patients with LUAD was assessed. The findings indicated a significant correlation between the prognosis of patients with LUAD and the expression level of FSTL3 (HR = 0.4, 95%CI 0.2–0.8, *p* = 0.01) (Fig. 6G). Immunohistochemistry results indicated a significant increase in the expression of FSTL3 in fatal cases (*p* < 0.0001). At the same time, we analyzed the basic data of the 94 patients included in the study and drew baseline data tables (Table 5) and one-way COX regression analyses (Table 6). The results showed that the FSTL3 low-expression group and the high-expression group did not show statistically significant differences in age, gender and pathological grading. However, there were significant differences in clinical stage and survival time between the two groups. However, one-way COX regression analysis based on survival prognostic data showed that surviving patients and dying patients did not show significant differences in age, gender, pathological grading, and clinical stage.

**Table 5:** Characteristics between FSTL3 low-expression group and FSTL3 high-expression group in LUAD patients of validation dataset

Characteristics	FSTL3-low	FSTL3-high	*P* value
*n*	47	47	
Age, *n* (%)			1.000
60 ~	20 (21.3%)	20 (21.3%)	
~ 59	27 (28.7%)	27 (28.7%)	
Gender, *n* (%)			1.000
Female	22 (23.4%)	22 (23.4%)	
Male	25 (26.6%)	25 (26.6%)	
Stage, *n* (%)			**0.015**
I	24 (25.5%)	11 (11.7%)	
II	11 (11.7%)	13 (13.8%)	
III	12 (12.8%)	23 (24.5%)	
Grade, *n* (%)			0.367
G3	31 (33%)	35 (37.2%)	
G2	16 (17%)	12 (12.8%)	
OS, *n* (%)			**0.003**
1	26 (27.7%)	12 (12.8%)	
0	21 (22.3%)	35 (37.2%)

**Table 6:** Univariate characteristics in LUAD patients of validation dataset

Characteristics	Total (*N*)	Univariate analysis	
		Hazard ratio (95% CI)	*P* value
Age	94		
60 ~	40	Reference	
~ 59	54	1.095 (0.571–2.099)	0.785
Gender	94		
Female	44	Reference	
Male	50	1.198 (0.632–2.272)	0.580
Stage	94		
I	35	Reference	
II	24	1.195 (0.502–2.844)	0.687
III	35	1.589 (0.758–3.328)	0.220
Grade	94		
G3	66	Reference	
G2	28	0.697 (0.329–1.475)	0.345

## Discussion

FSTL3, encoded by the *FSTL3* gene that is targeted by novel chromosomal rearrangements, belongs to the FSTL protein family (Kralisch et al. 2017). In 1998, FSTL was identified in the B cells of individuals with chronic lymphocytic leukemia (Hayette et al. 1998). Recently, FSTL3 has been shown to regulate numerous BP such as cellular differentiation, aging, obesity development, atherosclerosis progression, and tumor progression (Hayette et al. 1998; Sidis et al. 2002, 2005). FSTL3 also plays a role in the development of different types of malignancies, including hepatocellular carcinoma (Grusch et al. 2006), breast cancer (Bloise et al. 2009), and NSCLC (Nishimura et al. 1982). Tumor development is heavily regulated by FSTL3. In this study, we investigated FSTL3 expression and its prognostic significance in LUAD by utilizing TCGA database, our center's validation dataset, the TIMER database, and R software. In both TCGA and our validation set datasets, FSTL3 expression was notably reduced in tumor tissues compared to that in normal tissues. Furthermore, increased FSTL3 expression was linked to unfavorable prognosis in patients with LUAD. Patients with LUAD and exhibiting elevated levels of FSTL3 expression demonstrated a higher propensity for lymph node metastasis and presented with inferior clinicopathological staging compared to LUAD patients with lower FSTL3 expression. GSEA, GO, and KEGG pathway enrichment analyses were used to investigate the possible biological roles of FSTL3. This analysis revealed that FSTL3 expression was associated with nuclear division and spindle and microtubule binding. We then further examined the association between FSTL3 expression and prognosis as well as immune components in LUAD. Furthermore, immunohistochemistry was used to investigate the protein expression levels of FSTL3 in an external validation cohort at our institution. Overall, the findings of this research indicate that FSTL3 shows potential as a predictive marker for LUAD.

Tumor biology studies have increasingly focused on the relationship between tumors and the tumor microenvironment (TME) in recent years, which is crucial for understanding tumor pathogenesis and the effectiveness of immunotherapy (Chakravarthy et al. 2018; Henke et al. 2019). The effectiveness of immunotherapy in various forms of cancer is dependent on the TME. Nevertheless, LUAD presents a significant obstacle to personalized therapy due to its varied characteristics and unfavorable prognosis. The cells in the TME demonstrate flexibility by altering their antigenicity, evading immune detection, and persistent proliferation. Consequently, these cells can stimulate the activity of signal effectors, ultimately promoting the progression of tumors. Intratumor heterogeneity continues to pose a challenge in immunotherapy (Spella and Stathopoulos 2021). Cancerous and host cells in LUAD tumors form a diverse environment containing inflammatory signals, including cytokines, chemokines, and their corresponding receptors (Allavena et al. 2011). Activation of this inflammation signaling results in the recruitment of different types of immune cells, such as macrophages associated with tumors, lymphocytes that are reactive to tumors, suppressor cells derived from myeloid cells, neutrophils associated with tumors, and mast cells. These immune cells interact with tumor cells, leading to the development of a highly immunosuppressive TME. This environment reduces the effectiveness of immunotherapy to kill tumor cells and promotes tumor growth (Bronte et al. 2006; Zaynagetdinov et al. 2011; Ostrand-Rosenberg et al. 2012). Conversely, within the TME, the arrangement and role of infiltrating immune cells in tumors can exhibit minor differences based on the stage of the tumor and the immune condition of the host (Xiong et al. 2018).

In this study, we examined the relationship between FSTL3 and immune infiltration in LUAD and found a significant correlation between the expression of FSTL3 and B cells, CD4 + T cells, macrophages, neutrophils, and CD8 + T cells, indicating that the expression of FSTL3 is associated with the presence of immune cells in LUAD tissues. Various types of immune cells, such as Th1 cells, Tregs, CD8 + T cells, DC, macrophages, neutrophils, and NK cells, were present in the tumor immune microenvironment of LUAD tissues. The expression of FSTL3 was significantly correlated with the expression of marker genes of various immune cells. Correlation analysis revealed a significant association between FSTL3 expression and B cell infiltration, as well as markers of different types of T cells, demonstrating a strong correlation between FSTL3 expression and the infiltration of immune cells in LUAD.

After analyzing genes that showed positive and negative correlations with FSTL3 expression, we found that alterations in the expression of six genes, *KRT6A*, *VEGFC*, *KRT14*, *SNORD17*, *SNORA12*, and *KRT81*, had a notable impact on the prognosis of patients with LUAD. These findings may guide future studies on the molecular mechanisms associated with the impact of FSTL3 on the prognosis of patients with LUAD.

Our investigation revealed a notable diminution in FSTL3 expression among lung adenocarcinoma patients, which exhibited a significant correlation with N stage, pathological stage, and overall survival metrics. Moreover, we observed associations between FSTL3 levels and the presence of B cells, T cells, NK cells, and neutrophils. These findings imply that FSTL3 potentially exerts its influence by modulating the tumor microenvironment, especially in terms of immune cell infiltration. Gene Set Enrichment Analysis further elucidated a strong linkage of FSTL3 with critical signaling pathways, notably those involved in DNA replication and cell cycle regulation. This underscores the pivotal role of FSTL3 in cellular proliferation and survival, thereby impacting tumor initiation and progression.

Looking forward, we aim to implement animal model experiments to substantiate the function and mechanisms of FSTL3 in lung adenocarcinoma more robustly. We plan to investigate the impact of FSTL3 downregulation or overexpression on tumor growth, metastasis, and immune evasion in lung adenocarcinoma. Furthermore, the prospect of FSTL3 as a viable target for immunotherapy is a primary focus of our future research. Considering the association of FSTL3 with diverse immune cell infiltrates, we intend to assess the therapeutic efficacy of immunotherapy strategies that target FSTL3 in treating lung adenocarcinoma. Concurrently, we will delve into the interactions between FSTL3 and other pertinent genes, such as KRT6A, VEGFC, KRT14, KRT17, SNORA12, and KRT81, to enhance our understanding of its role in lung adenocarcinoma progression and to pinpoint novel therapeutic targets. However, our study has certain limitations. We examined pertinent genes and molecular mechanisms using sequencing data from public databases, which require validation through future studies involving in vivo and ex vivo experiments.

In summary, FSTL3 expression is crucial for the prediction of LUAD prognosis and plays a crucial role in the progression of LUAD by regulating the infiltration of immune cells such as T lymphocytes and B lymphocytes in the TME. The associated genes identified here could potentially serve as novel targets for future studies on the diagnosis and treatment of LUAD.

### Supplementary Information

Below is the link to the electronic supplementary material.

Supplementary file1 (PDF 467 KB)

## Data Availability

The datasets used during the present study are available from the corresponding author upon reasonable request.
