# Supplementary material for: FSTL3 is associated with prognosis and immune cell infiltration in lung adenocarcinoma
Source: J Cancer Res Clin Oncol. 2024 Jan 19;150(1):17. doi: 10.1007/s00432-023-05553-w (PMC10799152; doi:10.1007/s00432-023-05553-w)
Supplement: Supplementary file 1 — Supplementary file1 (PDF 467 KB) [file 432_2023_5553_MOESM1_ESM.pdf]

Journal: International Journal of Clinical Oncology

Title: FSTL3 is associated with prognosis and immune cell infiltration in lung adenocarcinoma

Xiangzhi Meng[a], Xiaojian Zhao[a], Boxuan Zhou[a], Weijian Song[a], Yicheng Liang[b], Mei Liang[a], Minjun Du[a], Jianwei Shi[a], and Yushun Gao[a]*

a Department of Thoracic Surgery, National Cancer Center/National Clinical Research Center for Cancer/Cancer Hospital, Chinese Academy of Medical Sciences and Peking Union Medical College, Chaoyang District, Panjiayuan, Nanli 17, Beijing 100021, People's Republic of China
b Department of Thoracic Surgery, Sun Yat-sen Memorial Hospital, Sun Yat-sen University, 107 Yanjiang West Road, Guangzhou, 510120, China

Xiangzhi Meng and Xiaojian Zhao contributed equally to this work

*Corresponding Author :
Yushun Gao
E-mail address: ysgaopumc@163.com
Telephone number: +86 010-87787130

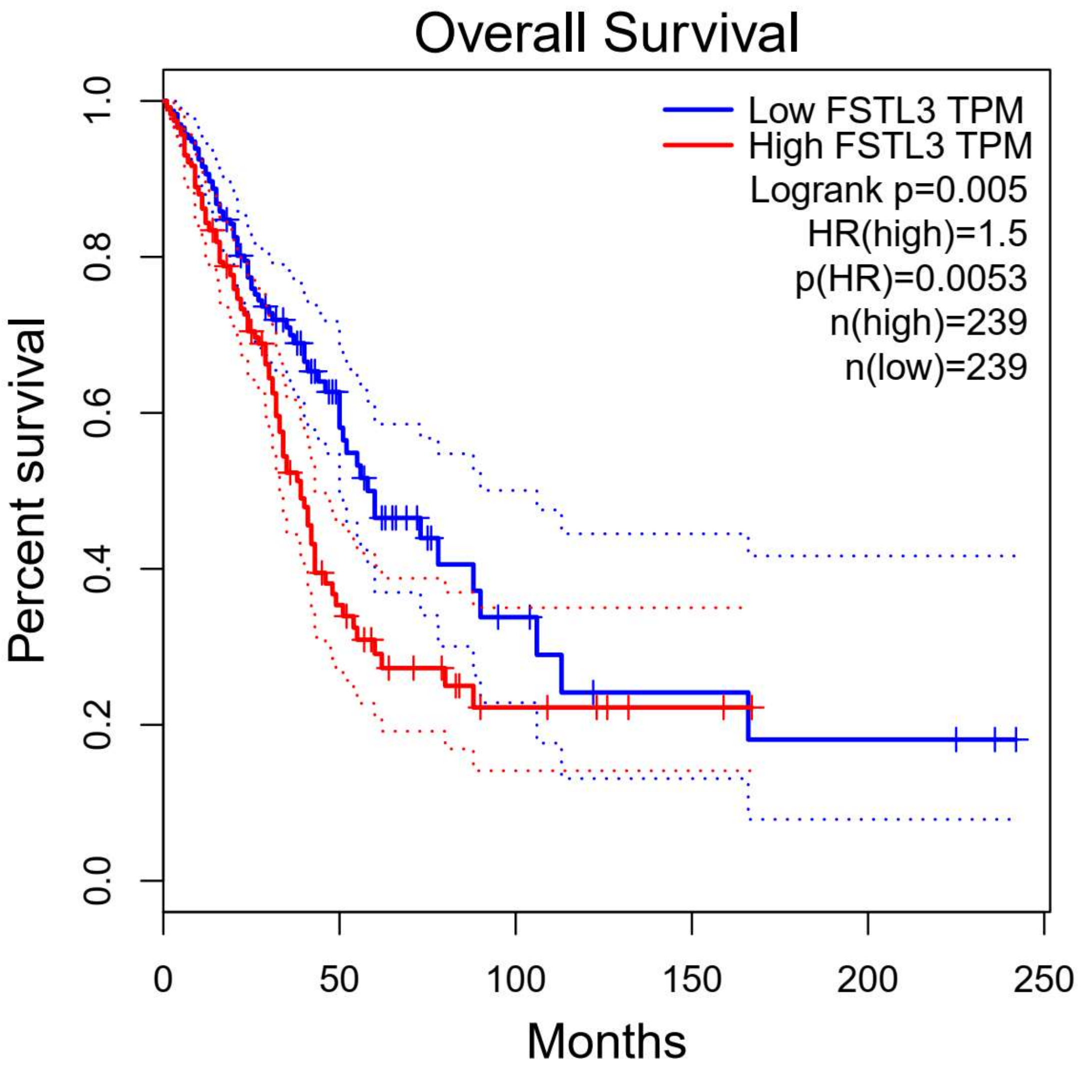

Supplemental Fig. 1 The analysis of overall survival (OS) and FSTL3 expression of patients with lung adenocarcinoma in the GEPIA online database

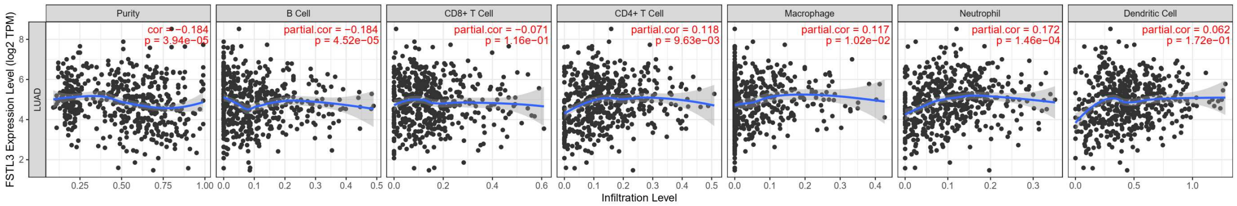

Supplemental Fig. 2 the correlation between FSTL3 expression and immune cell infiltration in tumor tissues in the TIMER online database

Supplementary table 1. the corresponding clinical characteristics of the patients in group according to FSTL3 expression

Characteristics	Low expression of FSTL3	High expression of FSTL3	P value
n	269	270	
Pathologic T stage, n (%)			0.484
T1	91 (17%)	85 (15.9%)	
T2	149 (27.8%)	143 (26.7%)	
T3	20 (3.7%)	29 (5.4%)	
T4	8 (1.5%)	11 (2.1%)	
Pathologic N stage, n (%)			0.054
N0	188 (35.9%)	162 (31%)	
N1	41 (7.8%)	56 (10.7%)	
N2	29 (5.5%)	45 (8.6%)	
N3	1 (0.2%)	1 (0.2%)	
Pathologic M stage, n (%)			0.571
M0	182 (46.7%)	183 (46.9%)	
M1	11 (2.8%)	14 (3.6%)	
Pathologic stage, n (%)			**0.031**
Stage I	163 (30.7%)	133 (25%)	
Stage II	57 (10.7%)	68 (12.8%)	
Stage III	32 (6%)	52 (9.8%)	
Stage IV	12 (2.3%)	14 (2.6%)	
Primary therapy outcome, n (%)			0.876
PD	35 (7.8%)	36 (8%)	
SD	18 (4%)	20 (4.5%)	
PR	2 (0.4%)	4 (0.9%)	
CR	166 (37%)	168 (37.4%)	

Characteristics	Low expression of FSTL3	High expression of FSTL3	P value
Gender, n (%)			0.577
Female	141 (26.2%)	148 (27.5%)	
Male	128 (23.7%)	122 (22.6%)	
Race, n (%)			0.322
Asian	5 (1.1%)	3 (0.6%)	
Black or African American	32 (6.8%)	23 (4.9%)	
White	199 (42.2%)	210 (44.5%)	
Age, n (%)			0.254
<= 65	135 (26%)	122 (23.5%)	
> 65	125 (24%)	138 (26.5%)	
OS event, n (%)			**0.021**
Alive	186 (34.5%)	161 (29.9%)	
Dead	83 (15.4%)	109 (20.2%)	
